# Nerve Growth Factor Peptides Bind Copper(II) with High Affinity: A Thermodynamic Approach to Unveil Overlooked Neurotrophin Roles

**DOI:** 10.3390/ijms22105085

**Published:** 2021-05-11

**Authors:** Antonio Magrì, Diego La Mendola, Enrico Rizzarelli

**Affiliations:** 1 Institute of Crystallography, CNR, Via Gaifami 18, 95126 Catania, Italy; leotony@unict.it (A.M.); erizzarelli@unict.it (E.R.); 2 Department of Pharmacy, University of Pisa, Via Bonanno Pisano 6, 56126 Pisa, Italy; 3 Department of Chemical Sciences, University of Catania, Viale A. Doria 6, 95125 Catania, Italy

**Keywords:** copper, NGF, Ctr-1, Alzheimer’s, protein, peptidomimetic, potentiometry, stability constants, beta amyloid, metallostasis

## Abstract

Nerve growth factor (NGF) is a protein essential to neurons survival, which interacts with its receptor as a non-covalent dimer. Peptides belonging to NGF N-terminal domain are able to mimic the activity of the whole protein. Such activity is affected by the presence of copper ions. The metal is released in the synaptic cleft where proteins, not yet identified, may bind and transfer to human copper transporter 1 (hCtr1), for copper uptake in neurons. The measurements of the stability constants of copper complexes formed by amyloid beta and hCtr1 peptide fragments suggest that beta-amyloid (Aβ) can perform this task. In this work, the stability constant values of copper complex species formed with the dimeric form of N-terminal domain, sequence 1–15 of the protein, were determined by means of potentiometric measurements. At physiological pH, NGF peptides bind one equivalent of copper ion with higher affinity of Aβ and lower than hCtr1 peptide fragments. Therefore, in the synaptic cleft, NGF may act as a potential copper chelating molecule, ionophore or chaperone for hCtr1 for metal uptake. Copper dyshomeostasis and mild acidic environment may modify the balance between metal, NGF, and Aβ, with consequences on the metal cellular uptake and therefore be among causes of the Alzheimer’s disease onset.

## 1. Introduction

Copper performs several essential functions as a cofactor in many enzymes in the living systems and it is required for the development and function of the human brain [1,2,3]. In recent years, it has been shown that copper, analogously to zinc, plays a role as modulator of cellular signal transduction pathways [4,5,6]. Copper is stored in synaptic vesicles and is released by electrical depolarization in the synaptic cleft of glutamatergic synapses at concentration values that can reach 100 μM [7]. Copper may have inhibitory or stimulating effect on synaptic plasticity, affecting memory and learning processes although the mechanisms by which the metal performs these functions remain largely undefined [8,9]. The dual effect may be related to the metal binding by different proteins expressed in neurons and released in the synapses as beta-amyloid (Aβ) [10]. 

The actual biological function of Aβ is still unknown. The polypeptide may control copper efflux in the synapses and it has been demonstrated that the polypeptide improves memory formation [11], synaptic plasticity, and neuronal survival [12]. 

In particular, the monomeric form of Aβ activates the cyclic AMP response element-binding protein (CREB), which in turn promotes the transcription and release of the brain-derived neurotrophic factor (BDNF) a neurotrophin (NT) strongly involved in long term potentiation (LTP) and memory formation [13].

It is worth noting that it has been demonstrated that copper ions may modulate kinase signaling networks of neuronal tissues induced by neurotrophins [14]. The nerve growth factor (NGF) is the first discovered member of NTs family [15]. NGF is essential for the development, survival, and activity of neurons [16,17]. NGF initiates the signaling pathways through the binding to tropomyosin receptor kinase A (TrkA) triggering a signaling cascade up to the activation of CREB [18,19]. 

Copper ions enhance NGF functionality and the effect seems related to the presence of metal binding site in the N-terminus domain of NGF [20]. NGF(1-14), a peptide encompassing the first 14 residue of NGF, is able to bind copper ions and mimic whole protein signal transduction activating CREB [21]. The NGF interacts with TrkA as a non-covalent dimer, so a dimeric form of the peptide NGF(1–14) has been tested, showing higher activity than monomers in the release of BDNF [14]. 

NGF signaling pathways control also the post-translational modifications of the amyloid precursor protein (APP) and then Aβ production in neurons [22,23,24]. Moreover, the deprivation in NGF determines Aβ aggregation and tau hyperphosphorylation in Alzheimer’s disease (AD) [25] while NGF addition protects against cell death and toxicity triggered by Aβ but the underlying mechanism remains unclear [26]. On the other hand, astrocytes activated by Aβ stimulate NGF secretion, whose excess in turn causes the death of hippocampal neurons [27].

In the dynamic environment present at the synaptic cleft, a potential interconnection among copper, Aβ, and NGF, could be one of the key components of the memory formation process. 

Therefore, the dyshomeostasis of copper could be at the basis of Aβ and/or NGF dysfunctions; conversely, a malfunction in the metabolic pathways could negatively affect the normal influx of copper into neurons [28]. The consequences could lead to the development of the progressive neurodegenerative disorder such as Alzheimer’s disease (AD) [29,30]. 

In the brains of patients affected by AD, a high copper concentration, up to 0.4 mM, is localized in senile plaques composed of amyloid aggregates [31]; however, the involvement of copper in AD is still controversial [32]. Studies show copper deficiency in patients affected by AD, and consequently a need to enhance copper levels so to restore normal metal concentration [33,34]; differently, other experiments point to metal overload and consequent demand to reduce copper concentration [35,36]. 

Copper dyshomeostasis with an increase in the labile pool of metal and a parallel decrease in the copper bound to proteins or peptides is the main interpretation [37,38,39]. A local imbalance of copper between extra- and intra-cellular spaces may drive its binding to Aβ, which may result in formation of oligomeric fibrils, amyloid, or amorphous aggregates, depending on the metal ion/peptide molar ratio.

Aβ may play the role of handling copper cellular influx/efflux via binding metal and transfer it to human copper transporter 1 (hCtr1), the main protein responsible for Cu import into cells [40]. Studies carried out with the peptide model showed that a peptide encompassing the first 14 residues of hCtr1 binds copper ions with higher affinity than N-terminal Aβ peptides [41]. For this reason, it is relevant to know the affinity of Aβ towards copper ions and many studies have been carried out with different techniques to determine the coordination environment of copper complexes formed with Aβ [42,43]. 

The pH value is another environmental condition that influences Aβ aggregation and Aβ copper binding. The brain pH is imbalanced towards mild acidic condition in aging and patients with AD [44]. A lowering of pH favors Aβ aggregation and strongly alters the release of pro-inflammatory cytokine affecting the uptake of Aβ by microglial cells [45]. 

Pathologies, such as ischemic stroke, induce a local decrease of extracellular pH due to an inflammatory insult that is as supposed to be an early event in AD progression [46]. Furthermore, the pH influences copper complex speciation with Aβ and mild acidosis can promote conformational changes or reactive oxygen species production promoting Aβ aggregate precipitation [47].

NGF has been postulated as potential therapeutic agent for the treatment of AD and its side effects, as loss of memory, and taking into account that NGF can bind copper ions in the same spaces shared by Aβ, it may be of interest to determine its affinity for copper(II).

Potentiometry is a technique that determines the stability constant values with a certain accuracy and permits also detection of minor species. This experimental technique may be the ideal choice when conditions allow for its application that can be limited by some factors such as poor solubility of longer or hydrophobic peptides. For this reason, potentiometric measurements have been carried out on shorter and more soluble Aβ fragments, in particular the N-terminal ones where the copper binding sites are found [43]. 

**Figure 1:**
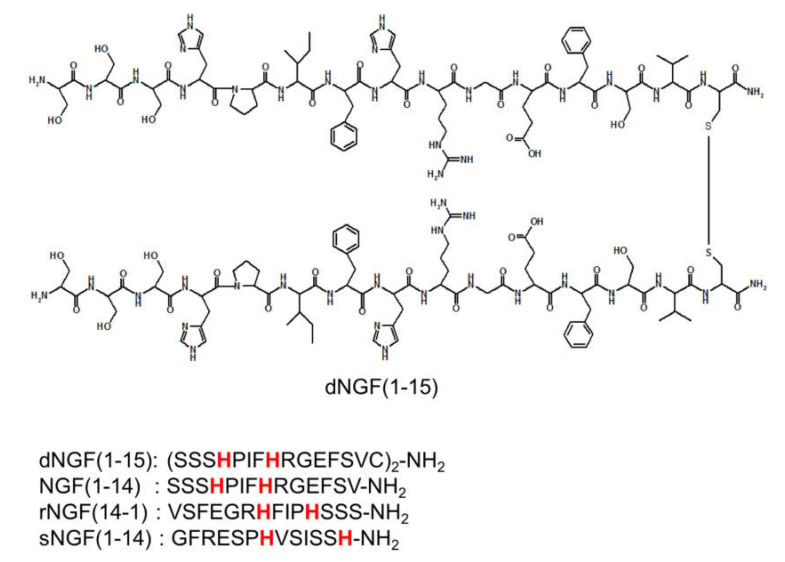
Structure of dNGF(1-15) and primary sequence of NGF N-terminal peptides. In red histidine residues that can act as metal anchoring sites.

In this paper, we report the stability constant values and copper coordination environment of a 30-mer peptide of NGF, namely the dimeric form of N-terminal domain 1-15 of the protein obtained by disulfide bridge between cysteine units in position 15 (Figure 1). The obtained data are compared to those reported for the monomeric peptide NGF(1-14) encompassing the N-terminal 14 residues of the NGF protein; the comparison of the affinity constants was extended to those obtained in this work on peptide encompassing the reverse rNGF(14-1) and another with a scrambled sNGF(1-14) sequence to highlight the role of primary sequence in copper binding. Finally, a comparison was made between the affinity constants obtained so far with those of the complexes formed by the copper(II) with Aβ and hCtr1 peptides. This comparison can be helpful to elucidate a molecular-level network among Aβ, NGF, and copper uptake by neurons which can alter synaptic activities under pathophysiologically relevant conditions.

## 2. Results

### 2.1. Protonation Constants

Peptides protonation constant values were determined by means of potentiometric titrations and are reported in Table 1 together with those of NGF(1-14) peptide. The monomeric ligands rNGF(14-1) and sNGF(1-14) show four proton accepting centers, as expected. The highest pK value corresponds to the N-terminal amino group. The protonation equilibria of the two histidine residues overlap and the average value for the protonation constant values of the imidazole residues is similar to that reported for analogous peptides [48,49]. The lowest pK value belongs to carboxylate side chain of glutamate and it agrees with that found for other peptides containing glutamic acid residues [50]. 

The dimeric peptide d(NGF1-15) was obtained by disulfide bridge between two units of the sequence 1–15 of the protein, exploiting the cysteine present in position 15. Therefore, the number of protonation sites is double compared to that of monomeric peptides, scrambled and reverse. The pK values of N-terminal groups are very similar to that of monomer NGF(1-14). Protonation reactions of the four imidazole side chains take place in completely overlapping reactions and the measured pK values are in a range between 6.7 and 6.2. The pK value of one carboxylate is the same of monomer peptide NGF(1-14) whereas the other carboxylate is significantly less acidic with a pK of 5.28.

### 2.2. Speciation and Characterization of Copper-Peptide Complexes

**Figure 2:**
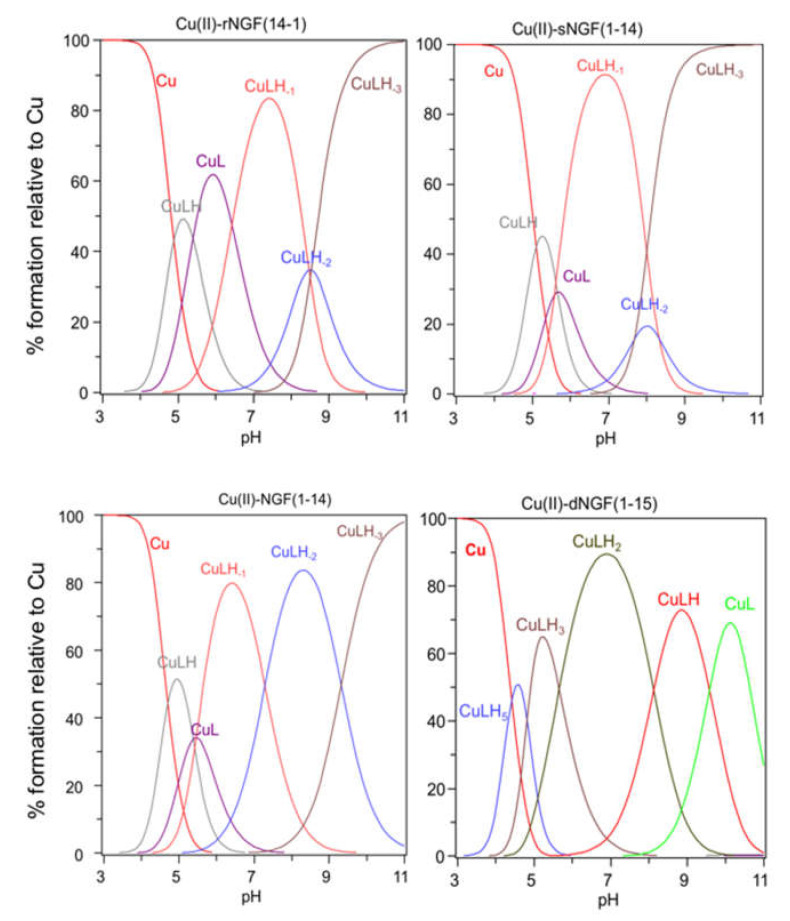
Species distribution of copper(II) complexes with rNGF(14-1), sNGF(1-14), NGF(1-14) and dNGF(1-15). [L] = 1 × 10^−3^ M; metal to ligand molar ratio of 1:1.

The stability constants of the Cu^2+^ complexes are listed in Table 2. The metal ion speciation for each peptide, at 1:1 metal-to-ligand molar ratio, is shown in Figure 2.

[CuLH] is the first complex species formed by rNGF(14-1) and sNGF(1-14) and reaches its maximum percentage of formation at pH 5 (Figure 2). The calculated stability constant values (logK_(111)_ = logβ_(111)_ − logβ_(011)_) are 6.33 and 6.12, for rNGF(14-1) and sNGF(1-14) respectively; the value obtained for reverse sequence is more similar to that of NGF(1-14) (logK_(111)_ = 6.53). The logK(111) values are in the range 6.0–6.5, then higher than those of similar Cu^2+^ complexes of peptide fragments in which the metal ion is bound to one imidazole nitrogen and one carboxylate [48,51,52]. Indeed, these values are in good agreement with those reported for analogous complex species formed by Aβ peptide fragments for which metal ions have been shown to form a macrochelate with a 2N_im_, COO coordination mode (log K = 6.18) [53,54].

Spectroscopic parameters measured at pH = 5, confirm the 2N_Im_,COO^−^ coordination mode for copper ion and the higher stability of metal complex formed by reverse (λ_max_ = 640 nm, ε = 64 M^−1^ cm^−1^) compared to scrambled peptide (λ_max_ = 670 nm, ε = 40 M^−1^ cm^−1^) (Table 3). However, it must be underlined that the parameters are partly influenced by the presence of other species other than free copper.

Increasing the pH, [CuL] complex species is formed. This species is a minor one but the obtained logβ value suggest the involvement of a further donor atom as N-terminal group for both peptides. The reverse peptide also shows for this species a higher value than scrambled one and very similar to that reported for NGF(1-14). 

The contemporary presence of an isomer in which a deprotonated amide is bound to metal instead of an amino group, that remains still protonated, cannot be ruled out.

**Figure 3:**
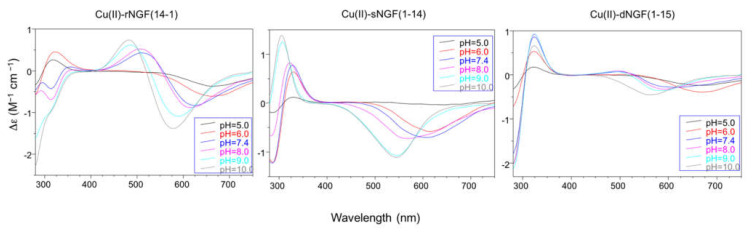
Circular Dichroism spectra with: rNGF(14-1), sNGF(1-14), and dNGF(1-15). [L] = 1 × 10^−3^ M; metal to ligand molar ratio of 1:1.

In particular the Cu-rNGF(14-1) system shows a CD signal centered around 320 nm, that is diagnostic of a charge transfer from a deprotonated amide nitrogen to copper ion [55] (Figure 3).

The next species formed, [CuLH_-1_], is the predominant complex in the pH range 6.5–8.0. The stepwise constant values logK_(11-1)_ (logK_(11-1)_ = logβ_(110)_ − logβ_(11-1)_) are 6.43 for rNGF(14-1) and 5.57 for sNGF(1-14). Both values indicate the deprotonation of an amide nitrogen atom but this step is more favored for sNGF(1-14). The UV–vis parameters are similar suggesting an analogous coordination environment of metal ion with three nitrogen atoms involved in the binding. CD spectra show the diagnostic signal of deprotonated amide nitrogen bound to copper ion even though the conformational features of two peptides are different due to distinctive primary sequence (Figure 3).

As the pH increases, the second deprotonation occurs but it is less favored than the third nitrogen amide deprotonation step. Indeed, the [CuLH_-2_] complex species is a minor one for both peptides, suggesting that the coordination of successive amide nitrogen deprotonation reactions are accompanied by the rearrangement of the peptide metal binding sites [56]. Namely, the histidine imidazole moiety and the subsequent amide are the primary binding sites below pH 8.5, but they are partly replaced by the amino group and preceding amide functions at higher pH values. This effect is supported by significant blue shift of the absorption spectra as well as of the CD band characteristic of peptide amino-bonded copper(II) complexes [57]. 

The first species formed by dimeric peptide dNGF(1-15) is [CuLH_5_] that reaches its maximum percentage at pH 4.5 (Figure 2).

The stability constant (logK_(115)_ = logβ_(115)_ -logβ_(015)_ = 41.18 − 35.12 = 6.06) is indicative of a 2Nim, COO^−^ coordination mode analogous to monomeric peptides and Aβ as above reported. This is further confirmed by UV–vis parameters similar to that of rNGF(14-1) copper complex. It is to note that in the [CuLH_5_] species there are only three deprotonated centers and therefore the involvement of 2N_im_ requires that one carboxylate group is still protonated. The unusual high pK value of one carboxylate moiety (pK = 5.28) and the pH range of species existence, between 4.0–5.5, makes this hypothesis plausible. 

[CuLH_3_] is the next species formed with a maximum percentage of formation around pH 5.5 (Figure 2). It is not possible to calculate the stepwise stability constant value as performed for other species because the protonation constant of the [LH_3_] species was not experimentally obtained. However, the difference with the stability constant of [CuLH_5_] (logK_(112)_ = logβ_(115)_ − logβ_(113)_ = 9.62) suggests the deprotonation of the carboxylate not coordinated to copper ion and the binding of another nitrogen atom (imidazole or amino) to the metal ion.

[CuLH_2_] is the predominant species at physiological pH; the stability constant value logK (logK_(112)_ = logβ_(112)_ − logβ_(012)_ = 10.56) is high and can be related with the relevant blue shift in the UV–vis maximum absorption (λ_max_ = 569 nm, ε = 94 M^−1^ cm^−1^). All these data are indicative of a strong ligand field around copper ion, determined by four nitrogen atoms coordination mode in a planar arrangement. It can be assumed that the (NH_2_, N^−^) five-membered chelate is assisted by the macrochelation with the N_Im_ donor of the His-4 residue of the same chain and the other histidine belonging to the other chain of dimeric peptide.

The CD spectra show an increase in the wide band centered at 324 nm, that include charge transfer signals of both imidazole and deprotonated amide to metal ion, and a d–d transition band with a relatively low intensity compared to the analogous species formed by monomeric peptides. These CD features are in agreement with the involvement of more imidazole side chain in the metal binding (Figure 3).

The next species [CuLH] and [CuL] show similar spectroscopic parameters to those of [CuLH_2_], suggesting the deprotonation of amino/imidazole not bound to metal ion and afterwards, at strongly basic pH, the deprotonation of an amide nitrogen atom that substitutes one imidazole in the coordination to metal ion.

## 3. Discussion

The peptide NGF(1-14) encompasses the first fourteen residues of the N-terminal domain of NGF protein. In a previous paper, we have demonstrated that NGF(1-14) and its dimeric derivative dNGF(1-15) form strong and significant interactions with TrkA, the specific NGF receptor, but not its reverse rNGF(14-1) nor the scrambled s-NGF(1-14) sequence [14]. However, in the presence of copper ions, both peptides NGF(1-14) and dNGF(1-15), induced signaling pathways independently by the receptor activation [14,21]. Conversely, the addition of copper has no effect on neuron cell models treated with rNGF(14-1) or sNGF(1-14) [14]. In a receptor-independent signaling processes, a different copper coordination environment may explain the absence of activity of scrambled and reverse sequence peptides compared to NGF(1-14) and dNGF(1-15).

Therefore, the peptides rNGF(14-1) and sNGF(1-14), designed to highlight the specificity of the primary sequence in the receptor recognition, can also unveil the critical role of the histidine residues with respect to the N-terminal amino group in the metal complex formation.

The protonation constants of two peptides rNGF(14-1) and sNGF(1-14) are similar to each other and to the values reported for the NGF(1-14). This indicates the absence of electrostatic bonds between the side chain groups. 

In a peptide, the pK range of protonation centers of the same type, for instance imidazole, widens as their number increases. A peptide encompassing three or more histidine residues shows a pK range of imidazole nitrogen atoms between 5.0–7.5 [58,59,60,61]. In the case of the dimeric peptide dNGF(1-15), the pK values determined for the amino and imidazole groups are very similar to those reported for the monomer peptides, indicating the absence of specific interactions between groups belonging to different peptidic chains. A different effect is observed only for the carboxylic groups: the monomer NGF(1-14) and the dimer dNGF(1-15) display a similar pK value for one carboxylate whereas a higher pK value is observed for the second carboxylate group of the dimer dNGF(1-15), indicating a marked reduction of acidity. This suggests a strong electrostatic interaction between the carboxylic anion of the first carboxylic group and the still protonated of the second one. 

At acidic pH, there is a certain similarity in the copper complex formation between the investigated peptides whereas at physiological pH, the metal displays a different coordination environment when bound to wild type NGF(1-14) and dNGF(1-15) in comparison with the reverse and scrambled sequences. 

In the complex species [CuLH] the copper ion displays a 2N_Im_,COO^−^ coordination mode for both rNGF(14-1) and sNGF(1-14). The reverse sequence peptide shows a higher copper complex stability constant of the macrochelate than sNGF(1-14); an effect due to the closer proximity of the two histidine residues and to the presence of a proline in between, that favors peptide bending and then the macrochelate formation (HFIPH vs. HVSISSH, for rNGF(14-1) and sNGF(1-14) respectively, see Figure 1).

Taking into account the higher number of protonation sites, [CuLH_5_] is the species formed by dNGF(1-15) analogous to [CuLH] detected for monomeric peptides. [CuLH_5_] is the first species formed by dNGF(1-15) and starts at a slightly lower pH compared to monomeric peptides, due to differences in protonation constant values. The spectroscopic parameters are similar to those reported for rNGF(14-1) and the stability constant is in agreement with a 2N_Im_,COO^-^ coordination mode but it is not possible to define if histidine residues involved in metal binding belong to the same chain or not. 

At physiological pH, the [CuLH_-1_] is the predominant species for both rNGF(14-1) and sNGF(1-14). The deprotonation step involve an amide nitrogen for both peptides but with a higher value for rNGF(14-1) compared to sNGF(1-14), 6.43 and 5.57, respectively. This difference suggests that the deprotonation does not occur at the amino terminus as in this case a similar value would be expected for the two peptides. Therefore, the deprotonation involves an amide of a histidine residue and the difference between rNGF(14-19 and sNGF(1-14) may be ascribed to proline amid the two histidine for reverse fragment (HFIPH). Indeed, the proline residue acts as a ‘break-point’ in copper ion coordination [62]. This results in a greater distortion of metal coordination plane for rNGF(14-1) as indicated by the high molar absorption coefficient value of UV–vis maximum absorption. Therefore, at physiological pH, copper bound to both scramble and reverse shows a different speciation and coordination environment compared to NGF(1-14) where the N-terminal amino group is involved in the metal binding [20]. In the wild type sequence, histidine is the fourth residue (His-4), then closer to the terminal amino group. Therefore, the sequence SSSH (see Figure 1) favors the simultaneous involvement of the amino and imidazole nitrogen atoms, prompting a greater stability of the copper complexes.

At physiological pH, [CuLH_2_] is the predominant species formed by dNGF(1-15). In this species the copper coordination is different from that observed for scrambled and reverse peptides. The stability constant value is higher and indicative of a strong ligand field around copper ion, confirmed by the difference in the UV–vis maximum absorption (λ_max_ = 569 nm) clearly more shifted towards blue compared to the species formed by rNGF(14-1) and sNGF(1-14). The difference observed is due to the involvement of the amino group in metal coordination so to determine a four nitrogen atoms (NH_2_, N^−^, 2N_im_) coordination mode in a planar arrangement. The amide deprotonation is more favored at Ser-2 due to five-membered chelate ring formation than at His-4 where the deprotonation process could form a six-membered chelate ring [63]. The stability constant of copper complex species formed by dNGF(1-15) is higher than that reported for monomer NGF(1-4). The presence of more histidine residues allows to involve the imidazole unit that determines a stronger coordination without inducing relevant distortions in peptide conformation.

This type of coordination is also confirmed by the ease with which successive species are formed, increasing pH. Indeed, the next deprotonation steps involve the side chains not directly involved in the metal binding, and, at a more basic pH, the amide nitrogen of Ser-3; this is further confirmed by the CD spectra that display the same conformational features even at increasing pH values.

It is to note that dimeric peptide does not form species with two copper atoms, confirming that amide deprotonation occurs in one of the two peptidic chains while the other one contributes with the coordination of one or two side chain imidazole groups to the overall stability of the copper complexes. 

The determination of the copper complexes stability constants formed by peptides of biological interest is a parameter that provides the affinity of a metal ion for a ligand on a quantitative basis. This can give insights for understanding the activity of such systems, while bearing in mind that the biological matrix is more complex and not an easy task [64].

However, the direct comparison between the stability constant values cannot provide the indication on which peptide binds metal ions more strongly since, as discussed above, they can form complex species with different deprotonation state [65]. For this reason, the apparent dissociation constant, $K_{D}^{app}$, is often used to describe, in the biological field, the affinity of a metal to a protein or a peptide. The $K_{D}^{app}$ is associated with equilibrium

(1) ML ↔M+L

and it is given by

(2) $$K_{D}^{app}=\;\frac{\left[M\right]\;\left[L\right]}{\left[ML\right]}$$

Therefore, the $K_{D}^{app}$ provides an overall data on the affinity obtained from the mean of the dissociation constants of all the species present in solution regardless of their stoichiometries and structures, at a specific pH value [43,66]. 

At pH 7.4, the $K_{D}^{app}$ for copper complexes formed by dNGF(1-15) and NGF(1-14) are higher than those calculated for complexes of rNGF(14-1) and sNGF(1-14), confirming that the presence of a His closest to N-terminal group allows the formation of a more stable copper complex (Table 4). 

The affinity values for copper ions of a peptide encompassing the sequence 1-16 of Aβ, functionalized with a polyethylene glycol moiety (Aβ_1-16_-PEG) to increase peptide solubility, were calculated from reported stability constant values [54]. Analogously, the $K_{D}^{app}$ values for copper complexes formed by the N-terminal fragment of hCtr-1 were calculated from potentiometric data reported in literature [40].

The comparison with the $K_{D}^{app}$ of copper complex formed by Aβ1-16, shows that both NGF(1-14) and dNGF(1-15) bind a single copper ion with higher affinity than Aβ fragment but lesser than hCtr_1-14_. 

It has been hypothesized that Aβ can chelate copper ions in the synaptic cleft and act as an extracellular metal chaperone for its delivery to hCtr1 protein. The release of copper from Aβ should be driven by the higher affinity of hCtr1 [40,41]. In competition with Aβ, NGF could perform a similar activity transporting copper to hCtr1. On the other hand, NGF having a high affinity for copper binding may capture copper ions in the synaptic cleft for different aims as cellular metal uptake through different pathways or neurons protection from metal excess. It is worth noting that copper addition increases activity of NGF as well as those of its N-terminal mimicking peptides. The pivotal role of copper is evidenced also by a general inhibitory effect on NGF and related peptides signaling cascade, determined by the addition of the bathocuproine disulfonic acid, a copper chelating agent [14,21].

Differently, at acidic pH the Aβ_1-16_-PEG peptide shows a higher affinity compared to the investigated NGF peptides even though the difference with dNGF(1-15) constant value is minimal. The peptide hCtr1-14 displays higher affinity for copper ions also at pH 5.5, so Aβ may act as metal chaperon for copper uptake in this environmental condition. However, it worth noting that a slight acidic pH promotes Aβ fibrils aggregation, limiting the chelation of copper by polypeptide. The combinations of these factors may determine toxicity and insult to neurons [67,68]. 

## 4. Conclusions

Potentiometric experiments carried out on N-terminal NGF peptides show that they bind metal with high affinity. NGF peptides are able to bind only one copper ion, even though they have more copper anchoring sites, differently from Aβ polypeptide that form multinuclear complex species. Therefore, the molar peptide-to-metal molar ratio can determine a different speciation between components present simultaneously at the synapses.

The determination of affinity constant values for copper ion indicates that at physiological pH and equimolar concentration, NGF peptides can compete with Aβ fragment for copper binding. Therefore, NGF could play a role in controlling copper homeostasis in the synaptic space as suggested for Aβ. This finding may partially explain the activity of copper complexes formed by N-terminal NGF peptides, which are able to promote CREB phosphorylation, a process pivotal to memory formation [14]. 

A slightly more acidic pH would make Aβ polypeptide, the most suitable ligand for copper release to hCtr1 compared with NGF peptides. In this environmental condition, NGF activity related also to the presence of copper ions could be limited with potential consequences on neuron physiology.

## 5. Materials and Methods

### 5.1. Chemicals

The peptides VFSEGRHFIPHSSS-NH2, rNGF(14-1) and GFRESPHVSISSH-NH2 sNGF(1-14) the scrambled sNGF(1-14) were synthesized) with the amidated C-terminal and purified as previously reported [14]. The dimer dNGF(1-15) was purchased from CASLO (Kongens Lyngby, Denmark).

All other chemicals, of the highest available grade, were purchased from Sigma-Aldrich (Munich, Germany) and used without further purification.

### 5.2. Potentiometric Titrations

Potentiometric titrations were performed on automatic instrument Titrando 905. A combined glass-Ag/AgCl electrode (Metrohm, Herisau, Switzerland) was used. All measurements were carried out on 2.0 mL of samples aqueous solutions of samples kept under an argon atmosphere and at a temperature of 298 K by means of a thermostat. Other details on the electrode calibration have been previously reported [69]. The ionic strength was fixed at 0.1 M by adding KNO_3_. KOH 0.1 M was used to titrate solutions containing either the free peptide or the peptide with Cu^2+^. The peptide concentrations used were 1 × 10^−3^ M. At least four independent titrations were performed. At the beginning of each measurement, the pH value was adjusted to 2.4 by addition of HNO_3_ 0.2 M and measurements were carried out up to pH 11. Metal ion to ligand molar ratios between 0.9:1 and 2.2:1 were used. The experimental data were analyzed by using HYPERQUAD 2003 program [70] and species distribution as a function of pH was obtained by means of Hyss program [71].

### 5.3. UV–Vis and CD Measurements

UV–vis spectra were recorded at 298.0 ± 0.2 K, by using an Agilent 8453 spectrophotometer. All the solutions were freshly prepared using double distilled water. UV–vis spectra were acquired by using 1.0 × 10^−3^ M peptide concentration at 1:1 metal to ligand molar ratio. The results are reported as ε (molar adsorption coefficient).

CD spectra were obtained at 25 °C under a constant N_2_ flow on a Jasco model 810 spectropolarimeter at a scan rate of 25 nm min^−1^, resolution of 0.1 nm, path length 1cm. Calibration of the instrument was performed with a 0.06% aqueous solution of ammonium camphorsulfonate. Spectra were recorded as an average of five scans. The CD spectra of the copper(II) complexes on varying the solution pH were obtained in the 280–750 nm wavelength region. CD spectra were acquired by using 1 × 10^−3^ peptide concentration at 1:1 metal to ligand molar ratio. The results are reported as Δε (molar circular dichroism), calculated as Δε = [θ]/3298.2 [72].

## Figures and Tables

**Table 1:** Protonation constant (log βpqr) and pK values (T = 298 °K and I = 0.1 M KNO_3_) ^a^.

Species	NGF(1-14) ^b^	rNGF(14-1)	sNGF(1-14)	dNGF(1-15)
LH	7.56	7.82 (2)	7.85 (3)	7.65 (5)
LH_2_	14.13	14.51 (2)	14.65 (3)	15.35 (2)
LH_3_	20.14	20.60 (2)	20.87 (3)	-
LH_4_	24.44	24.88 (2)	25.11 (4)	28.77 (6)
LH_5_	-			35.12 (4)
LH_6_	-			41.31 (4)
LH_7_	-			46.62 (4)
LH_8_	-			50.75 (4)
				
pK COO^-^	4.13	4.28	4.23	4.13
pK COO^-^	-	-	-	5.28
pK His	6.01	6.09	6.22	6.21
pK His	6.57	6.69	6.81	6.35
pK His (×2)	-	-	-	(6.71 × 2)
pK NH_2_	7.56	7.82	7.85	7.65
pK NH_2_	-			7.70

^a^ Standard deviations (3σ values) are given in parentheses. ^b^ Reference [20].

**Table 2:** Stability constants (log βpqr) and pK values of copper(II) complexes ^a^.

Species (pqr) ^b^	logβ_pqr_ NGF(1-14)^c^	logβ_pqr_ rNGF(14-1)	logβ_pqr_ sNGF(1-14)	logβ_pqr_ dNGF(1-15)
CuLH_5_	-	-	-	41.18 (3)
CuLH_3_	-	-	-	31.56 (2)
CuLH_2_	-	-	-	25.91 (5)
CuLH	14.09	14.15 (1)	13.97 (4)	17.79 (5)
CuL	8.72	8.77 (1)	8.32 (5)	8.21 (4)
CuLH_-1_	3.27	2.33 (2)	−2.74 (3)	-
CuLH_-2_	−4.02	−6.15 (4)	−5.58 (8)	-
CuLH_-3_	−13.34	−14.67 (2)	−13.27 (4)	-
pK (n/m)				
pK (5/3)	-	-	-	4.81 × 2
pK (3/2)	-	-	-	5.65
pK (2/1)	-	-	-	8.12
pK (1/0)	5.37	5.38	5.65	9.57
pK (0/−1)	5.45	6.43	5.57	-
pK (−1/−2)	7.29	8.48	8.33	-
pK (−2/−3)	9.30	8.52	7.69	-

^a^ Standard deviations (3σ values) are given in parentheses; ^b^ pCu + qH + rL = Cu_p_H_q_L_r_; β_bqr_ = [Cu_p_H_q_L_r_]/[Cu]_p_[H]_q_[L]_r_; ^c^ Ref. [20]. Charges are omitted for clarity; pK(n/m) values reflect the pK value of copper(II) complexes; [L] = 1 × 10^−3^ M; molar ratio 1:1.

**Table 3:** Spectroscopic parameters of Copper (II) complexes.

Peptide	pH	UV-vis λ (nm) (ε (M^−1^ cm^−1^))	CD λ (nm) (Δε (M^−1^ cm^−1^)
rNGF(14-1)	5	640 (64)	280 (−0.30); 316 (+0.20); 670 (−0.27)
	6	625 (144)	280 (−0.40); 323 (+0.33); 671 (−0.44)
	7.4	609 (178)	280 (−0.30); 314 (−0.22); 352 (+0.07); 508 (+0.31); 625 (−0.60)
	9	532 (194)	280 (−1.30); 484 (+0.46); 589 (−0.79)
	10	522 (232)	280 (−1.80); 483 (+0.54); 577 (−1.00)
sNGF(1-14)	5	670 (40)	289 (−0.20); 328 (+0.12); 647 (−0.04)
	6	617 (94)	287 (−1.34); 328 (+0.68); 617 (−0.59)
	7.4	603 (102)	288 (−1.38); 329 (+0.82); 603 (−0.72)
	9-10	522 (141)	306 (+1.47); 544 (−1.13)
dNGF(1-15)	5	635 (50)	280 (−0.34); 321 (+0.19); 669 (−0.24)
	6	612 (70)	280 (−0.40); 323 (+0.33); 671 (−0.44)
	7.4	569 (94)	280 (−1.80); 324 (+0.92); 504 (+0.08); 611 (−0.30)
	8	561 (104)	280 (−2.04); 323 (+0.98); 496 (+0.11); 586 (−0.34)
	9	554 (119)	280 (−2.15); 324 (+0.98); 496 (+0.08); 587 (−0.38)
	10	530 (138)	280 (−2.14); 324 (+0.71); 563 (−0.46)

**Table 4:** Apparent dissociation constant values for copper(II) complexes.

Peptide	pH	$K_{D}^{app}$
NGF(1-14)	7.4	2.5 × 10^−11^
dNGF(1-15)	7.4	4.2 × 10^−11^
rNGF(14-1)	7.4	6.5 × 10^−10^
sNGF(1-14)	7.4	2.7 × 10^−10^
Aβ_1-16_-PEG ^a^	7.4	1.1 × 10^−10^
hCtr_1-14_^b^	7.4	1.0 × 10^−13^
		
NGF(1-14)	5.5	4.0 × 10^−6^
dNGF(1-15)	5.5	1.9 × 10^−7^
rNGF(14-1)	5.5	1.4 × 10^−5^
sNGF(1-14)	5.5	3.8 × 10^−5^
Aβ_1-16_-PEG	5.5	2.5 × 10^−7^
hCtr_1-14_	5.5	7.4 × 10^−8^

^a^ Reference [54]; ^b^ Reference [40].

## Data Availability

All data are available from the corresponding author on request.
